# Knowledge, Practices, and Awareness Regarding Out-of-operating Room Sedation Among Non-anaesthesia Health Professionals: A Questionnaire Study

**DOI:** 10.4274/TJAR.2025.252044

**Published:** 2026-04-15

**Authors:** Yaşar Gökhan Gül, Selçuk Alver, Burak Ömür, Ayşe Nurmen Akın, Birzat Emre Gölboyu, Bahadır Çiftçi

**Affiliations:** 1 İstanbul Medipol University Faculty of Medicine, Department of Anaesthesiology and Reanimation, İstanbul, Türkiye; 2 Biruni University Faculty of Medicine, Department of Anaesthesiology, İstanbul, Türkiye; 3 İzmir Katip Çelebi University Faculty of Medicine, Department of Anaesthesiology, İzmir, Türkiye

**Keywords:** Anaesthesiology, non-anaesthesia health professionals, outpatient anaesthesia, questionnaire, sedation

## Abstract

**Objective:**

The efficacy and safety of sedation administered by non-anaesthesia healthcare professionals should be evaluated within the framework of evidence-based protocols, and approaches should be adopted to ensure patient safety at the highest level. We aimed, with a scientific approach, to evaluate non-operating-room anaesthesia applications performed by non-anaesthesia health professionals in terms of patient safety, quality, and consistency, and to identify areas of deficiency.

**Methods:**

After obtaining ethical approval, a questionnaire was prepared to evaluate practitioners’ awareness of the anaesthesia and sedation processes administered to patients during procedures performed in their clinics. An electronic questionnaire (Google Form) was used to collect data.

**Results:**

This study revealed that non-operating-room sedation applications are widely practiced across various specialties in our country, but levels of knowledge and skill regarding these applications are not standardized. Extending in-service training, developing practical skills in managing complications, and using objective criteria for patient follow-up after sedation are of great importance for patient safety and clinical efficacy.

**Conclusion:**

Standardization of sedation practices can be achieved through multidisciplinary cooperation and the adoption of protocols based on current guidelines. In this context, it is recommended that structured training programs and clinical guidelines be established for non-anaesthesia healthcare professionals.

**Main Points**

• Faster discharge rates, reduced health expenditures, and increased comfort demands from patients and physicians are increasing demand for non-operating-room anaesthesia (NORA) applications.

• Sedation competence requires practitioners to have knowledge of, and skills in, airway management, hemodynamic monitoring, and drug titration.

• The efficacy and safety of sedation administered by non-anaesthesia healthcare professionals should be evaluated within the framework of evidence-based protocols and approaches should be adopted to ensure patient safety at the highest level.

• In our study, we aimed to evaluate, using a scientific approach, the NORA applications performed by non-anaesthesia health professionals in terms of patient safety, quality, and consistency, and to identify areas of deficiency.

## Introduction

Today, the rapid development of medical technologies has made it possible for physicians to diagnose health problems more rapidly and initiate treatment promptly. Pain and anxiety experienced by patients during non-operative diagnostic and treatment procedures are important problems that reduce patient comfort and delay patients’ return to daily life after the procedure. Faster discharge rates, reduction of health expenditures, and comfort demands of patients and physicians are increasing the demand for non-operating-room anaesthesia (NORA) applications.^1, 2, 3^

Demands by health professionals on anaesthesiologists have led to the emergence of new areas of practice. Sedation and analgesia are administered to patients during diagnosis and treatment in ear, nose and throat, dental, gastroenterology, psychiatry, cardiology, neurology, radiation oncology, and urology clinics.^4, 5, 6, 7^ Sedation procedures are also used in various non-operating-room settings, including complex imaging-guided interventions in radiology and diagnostic and therapeutic endoscopic procedures in gastroenterology.

The wide working area increases the workload of anaesthesia professionals and prevents anaesthesiologists from accessing all areas of the workspace. While national guidelines for sedation exist, they are primarily directed at anaesthesiologists. The regulations governing sedation administered by non-anaesthesia healthcare professionals are less well defined, creating a potential gap in standardized practice and patient safety.

Sedation competence requires practitioners to have knowledge and skills in airway management, hemodynamic monitoring and drug titration.^8, 9, 10^ The responsibility for anaesthesia and sedation administered by non-anaesthesia professionals during NORA applications rests with the physician performing the procedure. Therefore, the efficacy and safety of sedation administered by non-anaesthesia healthcare professionals should be evaluated within the framework of evidence-based protocols, and approaches should be adopted to ensure the highest level of patient safety.

In our study, it was aimed to evaluate the NORA applications performed by non-anaesthesia health professionals with a scientific approach in terms of patient safety, quality, and consistency and to identify the areas of deficiency.

## Methods

### Compliance with Ethical Standards

This survey study was approved by the Istanbul Medipol University Non-invasive Clinical Research Ethics Committee (approval no.:1006, decision number: 07.12.2023).

### Study Design

After obtaining ethical approval, the questionnaire questions were prepared to evaluate the awareness levels of the practitioners in the process of anaesthesia and sedation given to the patient during the procedures they performed in their clinics. An electronic questionnaire form (Google Form) was used to collect data. All participants consented to participation, data processing, and inclusion of their data in medical research at the beginning of the survey. Informed consent was obtained from the participants. The prepared questionnaire was distributed to assistants, specialists, and faculty members in the clinics at our hospital where anaesthesia and sedation are administered outside the operating room, via a WhatsApp link.

Participants were presented with an informative text in the introductory section of the questionnaire that described the purpose and nature of the study. The questions were determined by a consensus of the authors and were subsequently reviewed for content validity, clarity, and relevance by an expert panel consisting of (five senior anaesthesiologists and three senior physicians from procedural specialties) before distribution, and 16 questions were prepared to measure the duration of experience of the practitioners, their experience of anaesthesia and sedation in the procedures they frequently performed in their branch, the importance of preoperative evaluation, whether the physicians participated in in-service training, the complications they experienced and their causes, the discharge criteria after the procedure, and their awareness of the effects and side effects of anaesthetic and analgesic drugs. A questionnaire was sent to 150 practitioners, and data from the 118 practitioners who completed the questionnaire within the specified dates were analyzed. A formal sample size calculation was not performed because this was an exploratory, descriptive, questionnaire-based study. We used a convenience sample by sending the questionnaire to 150 practitioners.

The survey questions did not impose any age-group or specialty restrictions. Based on responses to all questions, the impact of sedation and analgesia applications performed outside the operating room by non-anaesthesia professionals on patient and operator safety was evaluated.

It is not known from which personal phone the link originated, and the confidentiality of personal information is maintained. Survey participants are informed about the protection of personal data before starting the survey. People who agree to participate in the survey are committed to volunteerism. Responses were collected and statistical findings were obtained. The survey questions are presented in Table 1.

### Statistical Analysis

The collected data were analyzed using SPSS 22.0 statistical software (IBM Corp., Armonk, NY, USA). Frequency distributions were calculated and presented as numbers and percentages.

## Results

In this study, a questionnaire form about sedation practices outside the operating room was delivered to 118 participants, and the responses of all of them were analyzed administered to 118 participants, and all responses were analyzed. Of the participants, 59.4% were male (n = 70) and 40.6% were female (n = 48). By educational status, 33 (27.9%) were residents, 16 (13.5%) were specialist doctors, 24 (20.3%) were assistant professors, 9 (7.6%) were associate professors, and 36 (30.5%) were professors.

**Figure 1:**
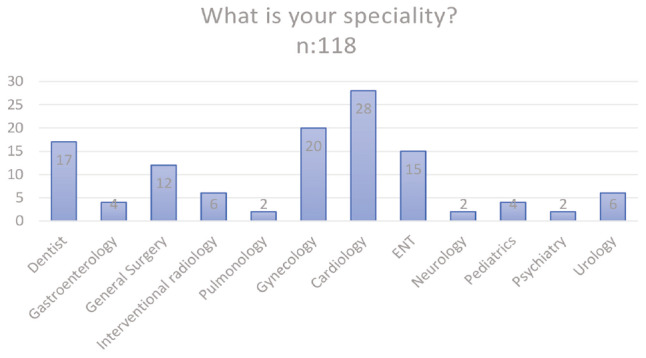
Specialization areas of the participants. ENT, ear, nose, and throat.

When the duration of professional experience of the participants duration of professional experience was analyzed, 29 (24.5%) had 0-5 years of experience 17 (14.1%) 6-10 years, 15 (12.7%) had 11-15 years, 9 (7.6%) had 16-20 years, and 48 (40.6%) had 21 years or more (Figure 1).

While 24.6% (n = 29) of the participants stated that they received in-service training on sedation practices outside the operating room, 72.8% (n = 86) stated that they did not receive training, and 2.5% (n = 3) stated that they had no opinion on this issue.

**Figure 2:**
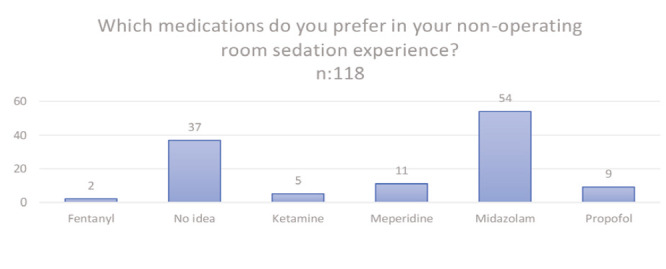
The sedative agents preferred by the participants.

When the perception of adequacy regarding drug doses was analyzed, 29 participants (24.6%) reported that the doses were adequate, 76 (64.4%) reported that the doses were inadequate, and 13 (11%) had no opinion on this issue (Figure 2).

While 74.5% (n = 88) of the participants reported having information about the fasting period before the procedure, 10.1% (n = 12) reported that they did not have information, and 15.2% (n = 18) were undecided.

**Figure 3:**
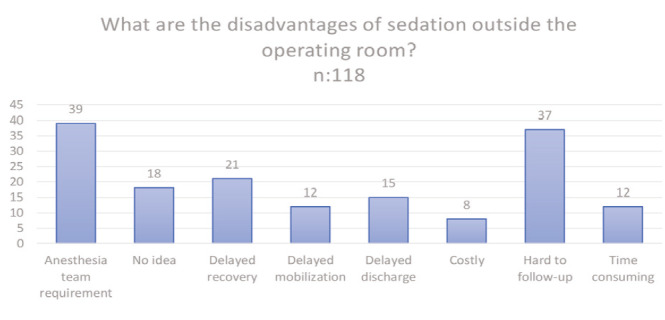
The disadvantages of sedation outside the operating room.

Of the participants, 23 participants (19.5%) reported experiencing medication-related complications, 75 (63.5%) did not, and 20 (16.9%) were unsure. Figure 3 illustrates the disadvantages of administering sedation outside the operating room. These reported complications were generally minor adverse drug reactions (e.g., nausea, vomiting, and dizziness) and did not necessitate a Code Blue call; this was assessed separately.

When the participants perception of competence in complication management was evaluated, 35 participants (29.6%) reported feeling competent, 68 (57.6%) reported not feeling competent, and 15 (12.7%) were undecided. While the specific reason for activation was not collected in the survey, Code Blue events during procedural sedation are most commonly initiated by life-threatening respiratory events (e.g., respiratory failure or apnea) or by profound cardiovascular instability (e.g., cardiac arrest or severe hypotension).

When the status of issuing a “Code Blue” alert was analyzed, 20 participants (16.9%) stated that they issued the alertreported issuing the alert, 82 (69.5%) did not, and 16 (13.5%) were undecided.

When asked about the use of assessment scales in discharge planning, only 6 participants (5%) reported using a scale, 79 (66.9%) reported not using one, and 33 participants (27.9%) were undecided.

Regarding the patient age-group they most frequently worked with, 16 participants (13.5%) worked with patients aged 0-8 years, 2 participants (1.6%) with patients aged 9-16 years; 60 participants (50.8%) with patients aged 17-64 years; 15 participants (12.7%) with patients aged 65+ years and older, and 25 participants (21.1%) were undecided.

In the case of regarding fear or anxiety about the possibility of complications prior to non-operating-room sedation, 28 people (23.7%) stated that they were concerned, 49 people (41.5%) reported not being concerned, and 41 people (34.7%) were undecided.

**Figure 4:**
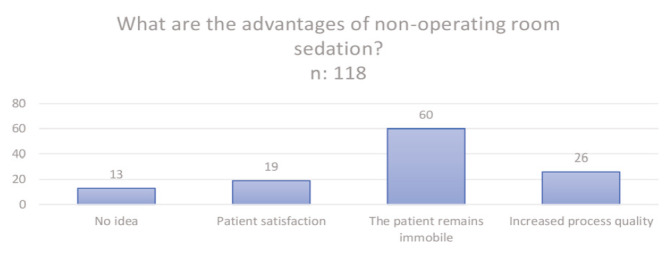
The advantages of sedation outside the operating room.

Figure 3 shows the disadvantages of sedation outside the operating room and Figure 4 illustrates the advantages of sedation administered outside the operating room.

When the time of sedation preference was questioned, when participants were asked about the timing of sedation, 46 (38.9%) stated that they preferred sedation uponat the patient’s request, 47 (39.3%) stated that they preferred sedation inthat they preferred sedation during complex procedures, 10 (8.4%) cited other reasons, and 14 (11.6%) were undecided. Figure 4 shows the advantages of sedation outside the operating room.

## Discussion

In this study, we evaluated physicians from different specialties regarding their knowledge, clinical practice habits, and complication-management competencies for sedation administered outside the operating room. The findings revealed that many participants lacked knowledge in certain areas necessary for the safe and effective administration of sedation.

Sedation is a widely used technique to ensure patient comfort and reduce anxiety and pain during medical and interventional procedures. This method, which can be applied in a spectrum ranging from light sedation to deep sedationa cross a spectrum from light to deep sedation, allows the patient to maintain helps the patient maintain hemodynamic stability by preserving the level of consciousness during the procedure. Appropriate management of sedation is critical to prevent complications such as respiratory depression, aspiration, and cardiovascular instability.^1, 2, 3, 4, 5^

To perform these procedures safely and effectively, it is important to determine appropriate sedation protocols and to evaluate the effectiveness of their application by non-anaesthesia health professionals.^11, 12^ Various complications related to a procedure or to anaesthesia may occur during the procedures.^11, 13, 14^ Patients should be thoroughly evaluated prior to the procedure to reduce complication rates. The properties of the anaesthetic and analgesic drugs to be used during the procedure should be well known; the patient’s tolerance and allergy status should be assessed and appropriate agents should be selected. Non-anaesthesia branch physicians who will perform the procedures should attend in-service sedation and analgesia training.^4, 5, 6, 7^

Only one-fourth of the participants (24.6%) reported receiving in-service training in sedation, highlighting the need for up-to-date guidelines and training programs.^1^ The higher rates of mortality and morbidity observed in patients receiving sedation in NORA settings compared with those in the operating room are primarily due to inadequate care and failures to adhere to safe practices.^2^ The American Society of Anesthesiologists emphasizes that standardization and quality control measures are essential to mitigate these risks in remote locations.^15^ Similarly, the proportion of participants who reported feeling competent in drug dosing and complication management was low. This situation raises potential risks to patient safety in sedation practices.^3^ Despite low self-perceived competence in drug dosage and complication management and a lack of formal training, non-anaesthesia professionals continue to perform these procedures. We speculate that this may be due to high clinical demand, pressure to increase throughput, and a shortage of anaesthesia professionals. We explicitly state that this discrepancy highlights a potentially significant risk to patient safety. Although 74.5% of the participants had information on the duration of fasting before the procedure, the fact that a significant proportion (25.5%) had insufficient information on this subject represents an important deficiency for preventing serious complications such as the risk of aspiration.^4^ That evaluation scales were not used extensively when making discharge decisions (66.9%) indicates that these decisions rely on subjective assessments. Complex interventions and patient demand stand out as the most common reasons for sedation. This finding indicates that sedation is not only a medical intervention but is also important for increasing patient satisfaction.^5^ Although monitored anaesthesia care implemented in non-operating-room settings increases patient comfort and procedural efficiency, safety considerations are also taken into account, thereby optimizing patient care.^6^ Approximately one-fifth of the participants (19.5%) reported drug-related complications, but the percentage who felt adequate in managing complications remained at 29.6%. This suggests that skills for intervening in complications should be improved.^7^ In sedation-induced airway management, early recognition and intervention are essential to prevent permanent sequelae. Keeping the necessary equipment and medications ready and accessible, knowing the management steps to be followed, and displaying them in the rooms, if necessary, are recommended to prevent complications.^10^ Ultimately, standardization of NORA practices will be possible only with multidisciplinary collaboration guided by published professional standards. The Anesthesia Patient Safety Foundation and other professional bodies have issued recent recommendations focusing on infrastructure, staffing, and continuous quality improvement necessary for the safe conduct of NORA.^16, 17, 18^

### Study Limitations

This study has some limitations. The sample size was small. A formal sample size calculation was not performed for this descriptive study; we used a convenience sample. On the other hand, the study was conducted at a single center. Multicenter and international studies would be beneficial to the literature. Finally, our survey asked only whether a “Code Blue” was called, not the specific reason for the activation.

## Conclusion

This study revealed that non-operating room sedation applications are widely practiced in various branches in our country, but the level of knowledge and skills regarding these applications is not standardized. Extending in-service trainings, developing practical skills in complication management, and using objective criteria in patient follow-up after sedation are of great importance in terms of patient safety and clinical efficacy.^8^

Standardization of sedation practices will be possible with multidisciplinary cooperation and adoption of protocols based on current guidelines. In this context, it is recommended to establish structured training programs and clinical guidelines for non-anaesthesia healthcare professionals.^9^

## Ethics

**Ethics Committee Approval:** This survey study was approved by the Istanbul Medipol University Non-invasive Clinical Research Ethics Committee (approval no.: 1006, decision number: 07.12.2023).

**Informed Consent:** Informed consent was obtained from the participants.

## Figures and Tables

**Table 1. Survey Questions:** 

What is your gender?
Your educational background?
Total length of professional service?
What is your specialty?
Have you received in-service training for sedation applications outside the operating room?
Do you feel adequate about the maximum and minimum doses of the drugs you use in your non-operating room sedation experience?
Which drugs do you prefer in your non-operating room sedation experience?
Do you have information about the fasting times to be observed before the procedure in your non-operating room sedation experience?
Have you experienced any complications during the procedure due to the medications used in your out-of-the-operating room sedation experience?
Do you feel competent in managing complications that may occur due to the medications you used in your out-of-the-operating room sedation experience?
Did you call a code blue in your out-of-the-operating room sedation experience?
Do you use any assessment scales when planning discharge after the procedure in your non-operating room sedation experience?
Which age range of patients do you work with the most in your out-of-the-operating room sedation experience?
Did you have any fears or concerns before out-of-the-operating room sedation?
What are the disadvantages of sedation outside the operating room?
What are the advantages of sedation outside the operating room?
When do you prefer to apply sedation?
